# Perceptions, Attitudes, and Barriers towards the Use of Central Board for Accreditation of Healthcare Institutions (CBAHI) Standards among Saudi Healthcare Providers

**DOI:** 10.3390/healthcare12020183

**Published:** 2024-01-12

**Authors:** Saeed M. Kabrah, Samer Abuzerr, Arwa Flemban, Layal Jambi, Ahmed Kabrah, Saad Alghamdi, Saeed M. Alghamdi, Ahmad A. Alshareef, Seham M. Melibary, Dakheelallah Homoud Al-Ghamdi, Najeeb O. Filfilan, Tahani S. Ralsan, Adel A. Alzhrani

**Affiliations:** 1 Department of Clinical Laboratory Sciences, Faculty of Applied Medical Sciences, Umm Al-Qura University, Makkah 24382, Saudi Arabia; amkabrah@uqu.edu.sa (A.K.); ssalghamdi@uqu.edu.sa (S.A.); 2 Department of Medical Sciences, University College of Science & Technology-Khan Younis, Gaza P.O. Box 8, Palestine; samer_516@hotmail.com; 3 Department of Social and Preventive Medicine, School of Public Health, University of Montreal, Montreal, QC H3C 3J7, Canada; 4 Pathology Department, Faculty of Medicine, Umm Al-Qura University, Makkah 24382, Saudi Arabia; aflemban@uqu.edu.sa; 5 Radiological Sciences Department, College of Applied Medical Sciences, King Saud University, Riyadh 11433, Saudi Arabia; ljambi@ksu.edu.sa; 6 Respiratory Care Program, Clinical Technology Department, Faculty of Applied Medical Sciences, Umm Al-Qura University, Makkah 24382, Saudi Arabia; smghamdi@uqu.edu.sa; 7 Laboratory and Blood Bank, PCR Department, AlNoor Specialist Hospital, Ministry of Health Makkah, Makkah 24241, Saudi Arabia; ahadalshareef@moh.gov.sa; 8 Quality Department, Regional Laboratory and Blood Banks, Ministry of Health Jeddah, Jeddah 22421, Saudi Arabia; smelibary@moh.gov.sa; 9 Health Support Services Centre, Ministry of Health Riyadh, Riyadh 12233, Saudi Arabia; dghamdi@moh.gov.sa; 10 Safety and Sterilization Department, Regional Laboratory and Blood Banks, Ministry of Health Makkah, Makkah 25215, Saudi Arabia; nfilfilan@moh.gov.sa; 11 Quality Management MOH Laboratory Accreditation Supportive CBAHI/CAP, Dammam 34116, Saudi Arabia; traslan@moh.gov.sa; 12 Quality Department, Regional Laboratory and Blood Banks, Ministry of Health Makkah, Makkah 25215, Saudi Arabia; adalzhrani@moh.gov.sa

**Keywords:** attitudes, barriers, CBAHI, healthcare providers, perceptions, Saudi Arabia

## Abstract

Background: Quality improvement is a strategic priority for all healthcare systems. However, the engagement of healthcare providers in pursuing accreditation plays a critical role in integrating standards into routine practice. Therefore, the current study assessed the perceptions, attitudes, and barriers towards using the Central Board for Accreditation of Healthcare Institutions (CBAHI) standards among Saudi healthcare providers. Method: This cross-sectional study was conducted in 2023 among a representative sample (364) of Saudi healthcare providers (both genders, aged 20–60) working at twenty governmental CBAHI-accredited hospitals in Saudi Arabia. The study participants were selected using a cluster random sampling method. Data regarding the perceptions, attitudes, and barriers toward using CBAHI standards among Saudi healthcare providers were evaluated using a validated questionnaire. Additional information regarding demographic–socioeconomic variables was obtained with an interview-based questionnaire. Statistical analysis was performed using SPSS version 28. Results: A total of 364 healthcare providers participated in the current study. Of them, 54.4% were males, and 45.6% were females. Almost half (48.6%) of the study participants held bachelor’s degrees. For the variables of age group, marital status, monthly income, and years of work experience, statistically significant associations were found between males and females (*p*-value < 0.05). The means of overall item agreement percentage of the participating healthcare providers for perceptions and attitudes towards using CBAHI standards and attitudes towards using CBAHI standards as a tool for quality improvement were 80.1%, 76.4%, and 72.0%, respectively. The highest item agreement percentage of the participating healthcare providers regarding the barriers that inhibit the hospital from obtaining the full benefit from the CBAHI accreditation was for the inexpedient IT tools (59.6%). Conclusion: The current study’s results demonstrated accepted perceptions and attitudes toward using CBAHI standards among Saudi healthcare providers. In addition, the identified barriers should be alleviated to improve the quality, effectiveness, and efficiency of the hospitals in Saudi Arabia. The findings also help clarify the accreditation operating process, which may be helpful to policymakers and stakeholders in making informed decisions on integrating accreditation standards.

## 1. Introduction

By enhancing the three major levels of structure, method, and outcome, accreditation is considered a tool to improve healthcare organizations’ quality, effectiveness, and efficiency [1]. Accreditation refers to the external peer review that evaluates a healthcare organization’s compliance with pre-defined performance standards, aiming to improve healthcare quality. Additionally, it is considered an internationally recognized methodical process that uses assessment and evaluating functions and practices to compare healthcare providers’ performance in specific geographic locations [2]. Over the past several years, the significance of employing accrediting procedures to improve healthcare facility efficiency has grown in Saudi Arabia [3].

The Central Board for Accreditation of Healthcare Institutions (CBAHI) is the official agency authorized to grant accreditation certificates to all governmental and private healthcare facilities operating in Saudi Arabia. It was established in 2005 to create and enforce quality standards in all Saudi Arabian healthcare provider organizations [4]. All hospitals licensed to practice in the Kingdom of Saudi Arabia are eligible for CBAHI accreditation, and eventually, all healthcare facilities operating in Saudi Arabia are required to achieve accreditation by CBAHI [5]. CBAHI sets the healthcare quality and patient safety standards against which all healthcare facilities are evaluated for evidence of compliance. The standards were developed by peer experts in the field and are of three major types depending on which area they are addressing: structure, method, and outcome [6,7]. Additionally, CBAHI offers healthcare facilities professional counseling, education, and training and shares the conclusions and recommendations of the analysis conditions wih the stakeholders [3].

All healthcare systems place a high focus on quality improvement. Additionally, certification may be a powerful tool for enhancing healthcare institutions’ services’ quality, efficiency, and sustainability over the long run [8]. By highlighting the areas of greatest need and expediting changes, accreditation initiatives can also result in better administration of hospital networks. A hospital’s ability to exhibit high levels of service delivery through accreditation can also favor performance in other healthcare sectors [9].

Healthcare and hospitals have been the subject of several quality improvement programs in Saudi Arabia, but providing efficient and long-lasting services has proven difficult [10]. The data on healthcare practitioners’ opinions of the effectiveness of certification paint a contrasting picture [11]. Accreditation has drawn criticism for being inefficient, time-consuming, expensive, bureaucratic, and insensitive to results in certain studies [12], while others have praised it for enhancing organizational performance and standardizing procedures [13,14].

Healthcare institutions’ ability to integrate standards is influenced by various context-specific elements. Critical among these is the healthcare practitioners’ interest in obtaining certification [15,16], which is closely tied to their perceptions—how they recognize and interpret the standards based on their previous experiences. Equally important is the willingness of healthcare practitioners to reflect on their attitudes—a settled way of thinking or feeling about certification, typically apparent in their behavior towards pursuing such professional endorsements. However, in the implementation of standards into everyday practice, barriers may occur—factors that impede or obstruct the pursuit of certification. Only a limited body of scientific research has examined perceptions, attitudes, and barriers regarding using CBAHI standards among Saudi healthcare providers, despite the widespread implementation of CBAHI accreditation in several hospitals across Saudi Arabia and the consensus that accreditation is linked to variables influencing the successful quality of healthcare and hospital outcomes. Hence, this study aims to fill this gap by assessing the perceptions and attitudes of Saudi healthcare providers towards CBAHI standards and identifying concerns and barriers to effective accreditation, thus contributing to a more nuanced understanding of the accreditation process within the Saudi Arabian healthcare context.

## 2. Materials and Methods

### 2.1. Study Design, Period, and Setting

A quantitative cross-sectional study was conducted between February and July 2023 at twenty governmental CBAHI-accredited hospitals in Saudi Arabia.

### 2.2. Ethical Approval

The current study was conducted after obtaining ethical approval from the Institutional Review Board at the Ministry of Health, Makkah, in Saudi Arabia (H-02-K-076-1022-828, 22 November 2022) and from King Saud University, College of Medicine, Institutional Review Board Committee (E-22-7096, 15 December 2022).

### 2.3. Eligible Population

Saudi healthcare providers (both genders, aged 20–60 years) working at twenty governmental CBAHI-accredited hospitals in Saudi Arabia were eligible to be included in the current study. These include paramedics, medics, and administrative staff in different Managerial positions.

### 2.4. Sample Size and Sampling Technique

In the current study, the representative sample of Saudi healthcare providers working at twenty governmental CBAHI-accredited hospitals in Saudi Arabia was calculated using the Charan and Biswas formula, which is specific for the study design used in [17]. Since exposure to recurring accreditation visits might influence the perception and attitudes of hospital healthcare providers and hence affect the validity of the findings [18], the inclusion was limited to hospitals that had one accreditation visit and had subsequently been accredited for at least six months before the study data collection process. The publicly accessible list of accredited hospitals on the CBAHI website revealed that twenty hospitals satisfied the inclusion criteria, from which a representative sample of Saudi healthcare providers was selected [19]. A total of 364 Saudi healthcare providers (both genders, aged 20–60 years) were determined using a cluster random sampling method.

### 2.5. Data Collection

Data on perceptions, attitudes, and barriers to using CBAHI standards among Saudi healthcare providers were evaluated using a validated questionnaire by six experts from relevant fields and pilot testing. The online questionnaire was distributed through various mediums, such as email, social media, and quality and accreditation officers. Additional information regarding demographic–socioeconomic variables was obtained with an interview-based questionnaire. Several strategic approaches were employed to optimize response rates and mitigate response bias in our survey. Primarily, the authors respected respondents’ time by designing a concise, focused questionnaire, eliminating extraneous questions, and ensuring language appropriateness for the target audience. Personalized survey templates were used to enhance engagement and relevance. Gentle reminders to nudge respondents toward completion were used to minimize response bias. Furthermore, the timing of the survey was carefully chosen to align with the respondents’ availability and relevance, focusing on active participants to enhance the survey’s effectiveness and reliability.

### 2.6. Questionnaire

Quantitative data was gathered using a questionnaire developed and validated by the relevant literature to collect the desirable data. The questionnaire consisted of five sections, as follows:

1. Socio-demographic characteristics of the healthcare providers (7 items).
2. The perceptions of participating healthcare providers towards using CBAHI standards (19 items).
3. The attitudes of the participating healthcare providers towards using CBAHI standards for accreditation (19 items).
4. The attitudes of the participating healthcare providers towards using CBAHI standards as a tool for quality improvement (16 items).
5. The participating healthcare provider’s barriers towards using CBAHI standards (6 items).

The questionnaire was prepared in the English language and then translated and administered in the Arabic language. Six experts from relevant fields ensured the questionnaire’s language appropriateness, content validity, question comprehensibility, and refinement before actual distribution among the participants. The questionnaire’s reliability, consistency, and stability were tested using the Cronbach alpha coefficient (α = 0.87).

### 2.7. Data Analysis

The Statistical Package for Social Science (SPSS, version 28) was used for data analysis. Descriptive statistics were used to describe continuous and categorical variables. Additionally, item agreement analysis was utilized in the study; it is a method that assesses the level of agreement among collaborating healthcare providers regarding their perceptions and attitudes toward implementing CBAHI standards. This method involved calculating the percentage of agreement for each item on the questionnaire and identifying items that fell below an acceptable agreement threshold. The choice of item agreement analysis was justified by its relevance to the study design, as it allowed for a detailed examination of the level of consensus or divergence on specific aspects related to CBAHI standards. Furthermore, the chi-square test was utilized to determine the statistically significant differences between categorical variables. A *p*-value of less than 0.05 was considered statistically significant.

## 3. Results

A total of 364 healthcare providers participated in the current study; 198 (54.4%) of them were males, and 166 (45.6%) were females. The participants were predominantly in the 31–45 age group (66.2%), followed by the 46–60 age group (23.1%) and the 20–30 age group (10.7%). A statistically significant difference was observed in the age distribution between genders (*p*-value = 0.040), with a higher proportion of males in the 46–60 age group (65.5%) compared to females (34.5%). The majority were married (266, 73.1%), with singles representing 19.8%. A significant gender difference was noted in marital status (*p*-value = 0.001), with higher proportions of single females (69.4%) and divorced females (95.8%). Participants’ educational levels varied, with the majority holding bachelor’s degrees (48.6%). Other educational attainments included Diploma (8.8%), Master (26.6%), Ph.D. (9.9%), and medical fellowship (6.0%). No significant gender difference was found in educational level (*p*-value = 0.977). Income distribution varied, with 26.1% earning more than SAR 20160. Significant gender differences were observed in income levels (*p*-value = 0.001), with a higher percentage of males in the highest income bracket (SAR >20,160). The sample included Physicians (23.9%), Nurses (41.5%), Pharmacists (3.3%), Radiologists and Radiological Technologists (6.9%), and Medical Laboratory Technicians (19.5%). No significant gender difference was noted in specialty (*p*-value = 0.173). Participants with more than 10 years of experience were the largest group (40.7%), followed by those with 1–5 years (35.4%) and 5–10 years (23.9%). There was a significant gender difference in work experience (*p*-value = 0.018), with a higher percentage of males having more than 10 years of experience. The distribution of managerial roles included Head of the Department (14.3%), Supervisor (24.2%), Director or Manager (13.2%), and those with no managerial position (48.4%). The difference in managerial roles between genders was not statistically significant (*p*-value = 0.073) (Table 1).

The average weight of overall item agreement percentage of the perception of the participating healthcare providers towards the use of CBAHI standards was 80.1%. The mean item agreement weight percentage of the respondents’ participation in the CBAHI accreditation was 73.1%. The average item agreement percentage of the benefits of the CBAHI accreditation was 84.4%. The average agreement weight of the quality outcomes of CBAHI accreditation was 79.7%. The perception of the participating healthcare providers towards the participation in the changes that resulted from accreditation recommendations of the CBAHI standards was high (100.0%), whereas the lowest item agreement weight was for the perception of the participating healthcare providers towards the recommendations as an opportunity to implement significant changes at the hospital (26.1%) (Table 2).

The mean of overall item agreement percentage of the attitudes of the participating healthcare providers towards using CBAHI standards for accreditation was 76.4%. The highest item agreement percentage was for the attitudes of the participating healthcare providers towards the use of CBAHI standards for accreditation as improving the reputation of the hospital (83.7%), whereas the lowest item agreement percentage was for the attitudes of the participating healthcare providers towards the using of CBAHI standards for accreditation as improving the hospital’s financial performance (61.5%) (Table 3).

The mean of the overall item agreement percentage of the attitudes of the participating healthcare providers towards using CBAHI standards as a tool for quality improvement was 72.0%. The mean of the total item agreement percentage of the perspectives of the participating healthcare providers towards the training of CBAHI standards as a tool for quality improvement was 64.7%.

The mean of the total item agreement percentage of the attitudes of the participating healthcare providers towards the inspection and report of CBAHI standards as a tool for quality improvement was 72.7%. The mean of the total item agreement percentage of the attitudes of the participating healthcare providers towards the policies and procedures of CBAHI standards as a tool for quality improvement was 78.4%. The mean of the total item agreement percentage of the attitudes of the participating healthcare providers towards the value for money of CBAHI standards as a tool for quality improvement was 51.4%.

The highest item agreement percentage was for the attitudes of the participating healthcare providers towards the CBAHI as leading to improved health and safety policies and procedures within the department (81.1%), whereas the lowest item agreement percentage was for the attitudes of the participating healthcare providers towards the CBAHI as leading to the department receiving a larger training budget (52.2%) (Table 4).

The highest item agreement percentage of the participating healthcare providers regarding the barriers that inhibit the hospital from obtaining the full benefit from the CBAHI accreditation was for the inexpedient IT tools (59.6%), whereas the lowest item agreement percentage was for the lack of relevance for their daily duties (43.7%) (Table 5).

## 4. Discussion

In the current study, the participants comprised healthcare professionals from a range of specialties, possessing diverse educational backgrounds, work experiences, and varying roles, including managerial positions. The sampling strategy employed was judicious, as it encapsulated a broad spectrum of healthcare providers. This diversity is instrumental in enhancing the generalizability of the study findings across a wider healthcare provider population. In addition, the significant differences in age, marital status, monthly income, and work experience between genders suggest these factors might influence healthcare providers’ perspectives and experiences in the context of CBAHI standards. This aligns with a study conducted in Saudi Arabia that found that gender, age, profession, department, and length of service were significantly associated with participation in accreditation and perceiving benefits of accreditation [3,20]. Another study also found that marital status can impact healthcare utilization, costs, and outcomes [21]. Conversely, the educational level may not be a major differentiator in the perception of accreditation standards [22]. The impact of managerial position on attitudes towards gender participation in the CBAHI study is insignificant, as attitudes towards accreditation are more influenced by organizational culture and personal values than by the hierarchical position [3,23]. Overall, demographic factors can play a role in shaping perceptions and attitudes toward CBAHI standards, and understanding these factors can help healthcare organizations tailor their approaches to accreditation and quality improvement.

The main findings of the current study revealed that the means of total item agreement percentages of the participating healthcare providers for perceptions and attitudes towards the use of CBAHI standards, attitudes towards using CBAHI standards as a tool for quality improvement, and barriers towards the using of CBAHI standards were 80.1%, 76.4%, 72.0%, and 54.1%, respectively. A small amount of scientific research has examined the perceptions, attitudes, and barriers to using CBAHI standards among Saudi healthcare providers, which made comparing the current results with previous studies difficult.

The majority of the healthcare professionals in the current study showed that the accrediting recommendations of the CBAHI standards were a chance to implement significant changes at the hospital. Still, all of them engaged in the changes that followed. In a prior study to gauge how Saudi Arabian medical professionals felt about hospital accreditation, Algahtani et al. found that accreditation had a positive influence on the process and implementation of change in the hospital, leading to improved patient care and other medical services [20]. For example, three cross-sectional studies undertaken in Iran, Denmark, and India investigating attitudes toward respective hospital accreditation programs concluded that accreditation was generally seen favorably, especially from the viewpoints of hospital managers and administrators [13,16,24].

Many healthcare professionals indicated that applying CBAHI criteria for accreditation enhanced the hospital’s reputation. At the same time, around half of them concurred that doing so enhanced the hospital’s financial performance. Furthermore, a significant portion of healthcare professionals agreed that CBAHI standards’ policies and procedures and their inspection and reporting could be helpful tools for quality improvement. At the same time, only about half of them thought using CBAHI standards could be a cost-effective quality improvement tool. Participants in a prior survey stated that participation in ongoing accreditation improves patient care, which reflects the organization’s reputation in today’s competitive marketplaces [25]. Aside from determining the cost of certification, which differed greatly between nations and programs, it was demonstrated that accreditation had a considerable positive impact on cost-cutting [26], increased the percentage of outpatient revenue [27], ensured higher productivity [28], and improved efficiency [29,30]; the results of the current study support these findings.

In addition, about two-thirds of the healthcare providers showed that using CBAHI standards has improved the department’s health and safety policies and procedures. In contrast, about half of them agreed that using CBAHI standards has led to the department receiving a larger training budget. Around two-thirds of the participating hospitals in significant cross-sectional research among 110 private hospitals in Lebanon evaluating the value of accreditation said that accreditation was an investment that was worthwhile since it improved the culture of quality and safety [31]. In actuality, direct costs were associated with the accreditation process, including hiring personnel and training healthcare professionals. The results of the current study align with those of a prior investigation on the influence of hospital accreditation on patient safety measures in Hail, Saudi Arabia, which discovered that accreditation improved the culture of incident reporting [32]. This result is consistent with a different investigation into Madinah hospitals’ understanding of the event reporting system [33]. The CBAHI accreditation program encouraged hospital employees to uphold the safety requirements established by the government, enhancing the quality of treatment and patient safety. The CBAHI requires hospitals to operate at the greatest safety standards, limiting medical mistakes and reducing recurrence rates [32].

The healthcare providers, on the other hand, demonstrated that the main obstacles preventing the hospital from fully benefiting from the CBAHI accreditation were distributed as follows: inefficient IT tools, a lack of time, a lack of support from the top administration, a lack of communication at the hospital/ward, a lack of support from the administration department, and a lack of relevance for daily tasks. Previous research has shown that the claimed worker shortage and hiring difficulties [34], infrastructure gaps, low levels of physician engagement [24], limited financial support [35], and bureaucratic decision-making procedures [36] represent barriers to accreditation standards implementation [37]. Further future studies are required to confirm these findings on a larger scale and eliminate selection bias.

The overall level of agreement among healthcare providers regarding CBAHI standards was found to be high. This can enhance perceptions and attitudes towards CBAHI accreditation and directly impact the quality level among healthcare providers in hospitals. However, a few items fell below the acceptable agreement threshold. These items can be addressed in future training and education programs. The findings of this study suggest that healthcare providers have positive perceptions and attitudes toward using CBAHI standards. However, there is still a need for further education and training to ensure that all providers are familiar with and can effectively implement CBAHI standards. By fostering a shared understanding and appreciation of CBAHI standards, educational and training programs can promote consensus and reduce variations in provider behavior. This, in turn, can lead to more consistent and effective implementation of CBAHI standards, ultimately improving patient safety and quality of care across Saudi Arabia’s healthcare system. The study’s primary limitation is its cross-sectional design, which restricts the generalizability of the present findings because it makes it impossible to establish a causal connection. The current study’s key advantages were that it was the first to examine Saudi healthcare professionals’ perspectives, attitudes, and impediments towards using CBAHI guidelines and its representative sample size.

## 5. Conclusions

This study comprehensively evaluates perceptions, attitudes, and barriers towards using CBAHI standards among healthcare providers in Saudi Arabia. The findings reveal a generally positive disposition towards the impact of CBAHI accreditation on quality improvement in healthcare settings. The overall agreement percentages regarding perceptions and attitudes towards CBAHI standards and their role in accreditation and quality improvement were substantial, indicating a recognition of the value these standards bring to healthcare practices. Significantly, healthcare providers acknowledged the role of CBAHI standards in enhancing hospital reputation and patient care quality. However, there was less consensus on the financial benefits of accreditation and its role as a cost-effective tool for quality improvement. This aspect underscores the need for further investigation into the economic implications of accreditation in healthcare.

The study also highlighted key barriers that healthcare providers face in fully leveraging the benefits of CBAHI accreditation, such as inefficiencies in IT tools, time constraints, lack of administrative support, and communication gaps within hospital settings. These findings resonate with global challenges in healthcare accreditation and emphasize the need for strategic approaches to address these barriers. While this study is pioneering in exploring Saudi healthcare providers’ views on CBAHI standards, its cross-sectional design limits the ability to draw causal inferences. Future research should aim to expand upon these findings through longitudinal studies and broader participant bases to validate these insights further and address any potential biases.

In conclusion, the insights from this study contribute valuable knowledge to the ongoing discourse on healthcare quality improvement in Saudi Arabia. They underscore the necessity of continued efforts to optimize the implementation of CBAHI standards, address identified barriers, and further explore the economic aspects of accreditation in healthcare. The current results pave the way for a deeper understanding of accreditation’s role in the Saudi healthcare system and its potential to enhance healthcare quality and safety.

## Figures and Tables

**Table 1:** Characteristics of the participating healthcare providers.

Variables	Total n (%) 364 (100)	Male n (%) 198 (54.4)	Female n (%) 166 (45.6)	*p*-Value
**Age (years)**				
20–30	39 (10.7)	17 (43.6)	22 (56.4)	0.040 *
31–45	241 (66.2)	126 (52.3)	115 (47.7)	
46–60	84 (23.1)	55 (65.5)	29 (34.5)	
**Marital status**				
Single	72 (19.8)	22 (30.6)	50 (69.4)	0.001 *
Married	266 (73.1)	175 (65.8)	91 (34.2)	
Divorced	24 (6.6)	1 (4.2)	23 (95.8)	
Widowed	2 (0.5)	0.0 (0.0)	2 (100)	
**Educational level**				
Diploma	32 (8.8)	18 (56.2)	14 (43.8)	0.977
Bachelor	177 (48.6)	94 (53.1)	83 (46.9)	
Master	97 (26.6)	54 (55.7)	43 (44.3)	
Ph.D.	36 (9.9)	19 (52.8)	17 (47.2)	
Medical fellowship	22 (6.0)	13 (59.1)	9 (40.9)	
**Monthly income (Saudi Riyal)**				
0–8699	47 (12.9)	20 (42.6)	27 (57.4)	0.001 *
8700–11,999	54 (14.8)	35 (64.8)	19 (35.2)	
12,000–15,299	84 (23.1)	32 (38.1)	52 (61.9)	
15,300–20,160	84 (23.1)	50 (59.5)	34 (40.5)	
>20,160	95 (26.1)	61 (64.2)	34 (35.8)	
**Specialty**				
Physician	87 (23.9)	57 (65.5)	30 (34.5)	0.173
Dentist	18 (4.9)	8 (44.4)	10 (55.6)	
Nurse	151 (41.5)	78 (51.7)	73 (48.3)	
Pharmacist	12 (3.3)	6 (50)	6 (50)	
Radiologist and Radiological technologist	25 (6.9)	10 (40)	15 (60)	
Medical laboratory technician	71 (19.5)	39 (54.9)	32 (45.1)	
**Work experience (years)**				
1 to 5	129 (35.4)	66 (51.2)	63 (48.8)	0.018 *
5 to 10	87 (23.9)	39 (44.8)	48 (55.2)	
More than 10	148 (40.7)	93 (62.8)	55 (37.2)	
**Managerial position**				
Head of the department	52 (14.3)	32 (61.5)	20 (38.5)	0.073
Supervisor	88 (24.2)	46 (52.3)	42 (47.7)	
Director or manager	48 (13.2)	33 (68.8)	15 (31.2)	
No position	176 (48.4)	87 (49.4)	89 (50.6)	

* The difference is significant at the 0.05 level (two-tailed).

**Table 2:** The perception of the participating healthcare providers towards the use of CBAHI standards. Perception is defined in this context as the healthcare providers’ recognition and interpretation of sensory stimuli, specifically the standards set by the CBAHI, based upon their previous experiences and interactions within the healthcare setting.

Variables	Strongly Disagree (%)	Disagree (%)	Neither Agree nor Disagree (%)	Agree (%)	Strongly Agree (%)	Item Agreement Percent (%)
**A: Items on respondents’ participation in the CBAHI accreditation**						
During the preparation for the last survey, important changes were implemented at the hospital						
Average weight (mean ± SD): 3.74 ± 1.4	56 (15.4)	27 (7.4)	0.0 (0.0)	152 (41.8)	129 (35.4)	77.2
I participated in the implementation of these changes						
Average weight (mean ± SD): 3.93 ± 1.3	37 (10.2)	47 (12.9)	0.0 (0.0)	100 (27.5)	180 (49.5)	77.0
I learned from the recommendations made to the hospital since the last survey						
Average weight (mean ± SD): 3.87 ± 1.2	44 (12.1)	9 (2.5)	0.0 (0.0)	208 (57.1)	103 (28.3)	85.4
These recommendations were an opportunity to implement important changes at the hospital						
Average weight (mean ± SD): 1.98 ± 1.3	211 (58.0)	58 (15.9)	0.0 (0.0)	78 (21.4)	17 (4.7)	26.1
I am participating in the changes that resulted from accreditation recommendations						
Average weight (mean ± SD): 4.66 ± 0.4	0.0 (0.0)	0.0 (0.0)	0.0 (0.0)	121 (33.2)	243 (66.8)	100
**B. Items related to the benefits of the CBAHI accreditation**						
Accreditation enables the improvement of patient care						
Average weight (mean ± SD): 4.08 ± 1.1	34 (9.3)	9 (2.5)	0.0 (0.0)	170 (46.7)	151 (41.5)	88.2
Accreditation enables the motivation of staff and encourages teamwork and collaboration						
Average weight (mean ± SD): 3.82 ± 1.0	14 (3.8)	49 (13.5)	0.0 (0.0)	224 (61.5)	77 (21.2)	82.7
Accreditation enables the development of values shared by all professionals at the hospital						
Average weight (mean ± SD): 3.70 ± 1.1	46 (12.6)	8 (2.2)	0.0 (0.0)	264 (72.5)	46 (12.6)	85.1
Accreditation enables the hospital to better use its internal resources (e.g., finances, people, time, and equipment)						
Average weight (mean ± SD): 3.58 ± 1.0	22 (6.0)	57 (15.7)	0.0 (0.0)	255 (70.1)	30 (8.2)	78.3
Accreditation enables the hospital to better respond to population needs						
Average weight (mean ± SD): 3.85 ± 1.1	24 (6.6)	38 (10.4)	0.0 (0.0)	208 (57.1)	94 (25.8)	82.9
Accreditation enables the hospital to better respond to its partners (other hospitals, diverse hospitals, private clinics, and others)						
Average weight (mean ± SD): 3.63 ± 0.9	15 (4.1)	49 (13.5)	0.0 (0.0)	289 (79.4)	11 (3.0)	82.4
Accreditation contributes to the development of collaboration with partners in the healthcare system						
Average weight (mean ± SD): 4.10 ± 1.1	16 (4.4)	48 (13.2)	0.0 (0.0)	117 (32.1)	183 (50.3)	82.4
Accreditation is a valuable tool for the hospital to implement changes						
Average weight (mean ± SD): 3.85 ± 0.7	0.0 (0.0)	41 (11.3)	0.0 (0.0)	295 (81.0)	28 (7.7)	88.7
Hospital participation in accreditation enables it to be more responsive when changes are to be implemented						
Average weight (mean ± SD): 4.26 ± 1.1	23 (6.3)	18 (4.9)	0.0 (0.0)	123 (33.8)	200 (54.9)	88.7
**C. Items on the quality of outcomes**						
Over the past three years, the hospital has shown steady, measurable improvements in the quality of customer satisfaction						
Average weight (mean ± SD): 3.78 ± 1.1	29 (8.0)	34 (9.3)	0.0 (0.0)	226 (62.1)	75 (20.6)	82.7
Over the past few years, the hospital has shown steady, measurable improvements in the administration’s quality of services (finance, human resources, etc.)						
Average weight (mean ± SD): 3.46 ± 1.0	14 (3.8)	83 (22.8)	0.0 (0.0)	254 (69.8)	13 (3.6)	73.4
Over the past few years, the hospital has shown steady, measurable improvements in the quality of care provided to patients (e.g., medical, surgical, obstetric, and pediatric patients)						
Average weight (mean ± SD): 3.98 ± 1.2	32 (8.8)	39 (10.7)	0.0 (0.0)	124 (34.1)	169 (46.4)	80.5
Over the past few years, the hospital has shown steady, measurable improvements in the quality of services provided by clinical support departments such as laboratory, pharmacy, and radiology						
Average weight (mean ± SD): 3.81 ± 1.1	21 (5.8)	48 (13.2)	0.0 (0.0)	205 (56.3)	90 (24.7)	81.0
Over the past few years, the hospital has maintained high-quality health services						
Average weight (mean ± SD): 3.76 ± 0.9	4 (1.1)	65 (17.9)	0.0 (0.0)	240 (65.9)	55 (15.1)	81.0
**The mean of overall item agreement percentages**						**80.1%**

**Table 3:** The attitudes of the participating healthcare providers towards using CBAHI standards for accreditation. In the scope of this study, ‘Attitudes’ are defined as the healthcare providers’ settled ways of thinking or feeling about the CBAHI standards for accreditation, which is typically reflected in their behavior and decision-making related to these standards.

Variables	Strongly Disagree (%)	Disagree (%)	Neither Agree nor Disagree (%)	Agree (%)	Strongly Agree (%)	Item Agreement Percent (%)
Standardizes care processes						
Average weight (mean ± SD): 4.07 ± 1.0	16 (4.4)	12 (3.3)	55 (15.1)	127 (34.9)	154 (42.3)	77.2
Increases quality of care by identifying problems						
Average weight (mean ± SD): 4.14 ± 1.0	16 (4.4)	14 (3.8)	38 (10.4)	128 (35.2)	168 (46.2)	81.4
Improves the health care level in the hospital						
Average weight (mean ± SD): 4.15 ± 1.0	14 (3.8)	15 (4.1)	43 (11.8)	122 (33.5)	170 (46.7)	80.2
Improves care in Saudi Arabia						
Average weight (mean ± SD): 4.12 ± 1.0	17 (4.7)	15 (4.1)	46 (12.6)	115 (31.6)	171 (47.0)	78.6
Improves patient safety						
Average weight (mean ± SD): 4.23 ± 1.0	14 (3.8)	12 (3.3)	38 (10.4)	112 (30.8)	188 (51.6)	82.4
Improves work environment						
Average weight (mean ± SD): 3.98 ± 1.1	22 (6.0)	20 (5.5)	61 (16.8)	99 (27.2)	162 (44.5)	71.7
Improves healthcare quality in my hospital/department						
Average weight (mean ± SD): 4.15 ± 1.1	23 (6.3)	11 (3.0)	36 (9.9)	112 (30.8)	182 (50.0)	80.8
Increases patient satisfaction						
Average weight (mean ± SD): 3.98 ± 1.1	25 (6.9)	15 (4.1)	58 (15.9)	109 (29.9)	157 (43.1)	73.0
Enhances the professional development of the staff						
Average weight (mean ± SD): 4.01 ± 1.1	24 (6.6)	17 (4.7)	59 (16.2)	93 (25.5)	171 (47.0)	72.5
Enhances feedback from the staff to the administrators						
Average weight (mean ± SD): 3.95 ± 1.2	23 (6.3)	22 (6.0)	66 (18.1)	90 (24.7)	163 (44.8)	69.5
Clarifies roles in the hospital						
Average weight (mean ± SD): 4.18 ± 1.0	15 (4.1)	11 (3.0)	46 (12.6)	113 (31.0)	179 (49.2)	80.2
Clarifies responsibility in the hospital						
Average weight (mean ± SD): 4.18 ± 1.0	15 (4.1)	11 (3.0)	42 (11.5)	118 (32.4)	178 (48.9)	81.3
Improves the hospital’s financial performance						
Average weight (mean ± SD): 3.78 ± 1.2	28 (7.7)	30 (8.2)	82 (22.5)	78 (21.4)	146 (40.1)	61.5
Improves the reputation of my hospital/department						
Average weight (mean ± SD): 4.30 ± 1.0	15 (4.1)	7 (1.9)	37 (10.2)	97 (26.6)	208 (57.1)	83.7
Supports the hospital administrative teams						
Average weight (mean ± SD): 4.09 ± 1.0	17 (4.7)	13 (3.6)	55 (15.1)	114 (31.3)	165 (45.3)	76.6
Supports the function of clinical teams						
Average weight (mean ± SD): 4.10 ± 1.1	17 (4.7)	17 (4.7)	50 (13.7)	107 (29.4)	173 (47.5)	76.9
Supports the function of interdisciplinary teams						
Average weight (mean ± SD): 4.06 ± 1.0	17 (4.7)	17 (4.7)	54 (14.8)	113 (31.0)	163 (44.8)	75.8
Supports internal cooperation by promoting the same norms and speech						
Average weight (mean ± SD): 4.06 ± 1.1	17 (4.7)	17 (4.7)	56 (15.4)	110 (30.2)	164 (45.1)	75.3
Supports the cooperation with healthcare staff across sectors						
Average weight (mean ± SD): 4.05 ± 1.0	16 (4.4)	17 (4.7)	60 (16.5)	109 (29.9)	162 (44.5)	74.4
**The mean of overall items agreement percentage**						**76.4%**

**Table 4:** The attitudes of the participating healthcare providers towards the use of CBAHI standards as a tool for quality improvement. In the scope of this study, ‘Attitudes’ are defined as the healthcare providers’ settled ways of thinking or feeling about the CBAHI standards as a tool for quality improvement, which is typically reflected in their behavior and decision-making related to these standards.

Variables	Strongly Disagree (%)	Disagree (%)	Neither Agree nor Disagree (%)	Agree (%)	Strongly Agree (%)	Item Agreement Percent (%)
**Training**						
CBAHI has led to the development of structured training for staff						
Average weight (mean ± SD): 3.91 ± 1.1	21 (5.8)	28 (7.7)	57 (15.7)	113 (31.0)	145 (39.8)	70.8
CBAHI has led to improved and more frequent updating of staff skills in terms of continuing professional development (CPD)						
Average weight (mean ± SD): 3.91 ± 1.1	24 (6.6)	23 (6.3)	58 (15.9)	115 (31.6)	144 (39.6)	71.2
CBAHI has led to the department receiving a larger training budget						
Average weight (mean ± SD): 3.50 ± 1.3	38 (10.4)	51 (14.0)	85 (23.4)	71 (19.5)	119 (32.7)	52.2
**The inspection and report**						
CBAHI has led to an improved reputation with service users						
Average weight (mean ± SD): 4.00 ± 1.1	21 (5.8)	21 (5.8)	57 (15.7)	101 (27.7)	164 (45.1)	72.8
CBAHI has led to more work being attracted to the hospital						
Average weight (mean ± SD): 3.87 ± 1.2	26 (7.1)	21 (5.8)	72 (19.8)	99 (27.2)	146 (40.1)	67.3
The CBAHI inspection was informative						
Average weight (mean ± SD): 3.97 ± 1.1	21 (5.8)	13 (3.6)	65 (17.9)	120 (33.0)	145 (39.8)	72.8
The existence of accessible, accurate, and updated guiding documents						
Average weight (mean ± SD): 4.13 ± 1.0	18 (4.9)	11 (3.0)	44 (12.1)	121 (33.2)	170 (46.7)	79.9
All members of the staff know of and work in coherence with relevant guiding documents						
Average weight (mean ± SD): 4.07 ± 1.0	15 (4.1)	18 (4.9)	51 (14.0)	119 (32.7)	161 (44.2)	76.9
Early detection of aggravation in disease						
Average weight (mean ± SD): 3.84 ± 1.2	25 (6.9)	24 (6.6)	73 (20.1)	103 (28.3)	139 (38.2)	66.5
**Policies and procedures**						
CBAHI has led to a change in procedures and/or policies within the department						
Average weight (mean ± SD): 4.06 ± 1.1	20 (5.5)	17 (4.7)	40 (11.0)	128 (35.2)	159 (43.7)	78.9
CBAHI has led to improved health and safety policies and procedures within the department						
Average weight (mean ± SD): 4.12 ± 1.0	19 (5.2)	15 (4.1)	35 (9.6)	127 (34.9)	168 (46.2)	81.1
CBAHI has improved the service delivered by the hospital						
Average weight (mean ± SD): 4.00 ± 1.1	21 (5.8)	16 (4.4)	66 (18.1)	98 (26.9)	163 (44.8)	71.7
Quality surveillance and external control a standard						
Average weight (mean ± SD): 4.14 ± 1.1	19 (5.2)	14 (3.8)	45 (12.4)	103 (28.3)	183 (50.3)	78.6
That requirements for quality improvement initiatives are a standard						
Average weight (mean ± SD): 4.15 ± 1.0	18 (4.9)	11 (3.0)	40 (11.0)	122 (33.5)	173 (47.5)	81.0
CBAHI has made the hospital focus more on quality standards						
Average weight (mean ± SD): 4.10 ± 1.1	22 (6.0)	12 (3.3)	42 (11.5)	117 (32.1)	171 (47.0)	79.1
**Value for money**						
The cost of CBAHI is value for money						
Average weight (mean ± SD): 3.56 ± 1.2	31 (8.5)	27 (7.4)	119 (32.7)	81 (22.3)	106 (29.1)	51.4
**The mean of overall item agreement percentages**						**72.0%**

**Table 5:** The participating healthcare providers’ barriers toward the use of CBAHI standards. Within this study, ‘Barriers’ are defined as factors that impede or obstruct healthcare providers’ pursuit of action or acceptance of CBAHI standards.

Variables	Strongly Disagree (%)	Disagree (%)	Neither Agree nor Disagree (%)	Agree (%)	Strongly Agree (%)	Item Agreement Percent (%)
**To what degree do you believe that the following barriers inhibit your hospital from obtaining the full benefit from the CBAHI accreditation**						
**Lack of time**						
Average weight (mean ± SD): 3.62 ± 1.1	19 (5.2)	53 (14.6)	78 (21.4)	111 (30.5)	103 (28.3)	58.8
**Inexpedient IT tools**						
Average weight (mean ± SD): 3.65 ± 1.1	18 (4.9)	52 (14.3)	77 (21.2)	108 (29.7)	109 (29.9)	59.6
**Lack of relevance for my daily duties**						
Average weight (mean ± SD): 3.30 ± 1.2	22 (6.0)	83 (22.8)	100 (27.5)	81 (22.3)	78 (21.4)	43.7
**Lack of communication at the hospital/ward**						
Average weight (mean ± SD): 3.52 ± 1.1	13 (3.6)	67 (18.4)	92 (25.3)	99 (27.2)	93 (25.5)	52.7
**Lack of support from the administration department**						
Average weight (mean ± SD): 3.51 ± 1.2	17 (4.7)	73 (20.1)	86 (23.6)	80 (22.0)	108 (29.7)	51.7
**Lack of support from the top administration**						
Average weight (mean ± SD): 3.66 ± 1.2	17 (4.7)	60 (16.5)	75 (20.6)	87 (23.9)	125 (34.3)	58.2

## Data Availability

The data presented in this study are available on request from the corresponding author.
